# The Added Value of Face-to-Face Supervision to a Therapeutic Exercise-Based App in the Management of Patients with Chronic Low Back Pain: A Randomized Clinical Trial

**DOI:** 10.3390/s24020567

**Published:** 2024-01-16

**Authors:** José Javier López-Marcos, María José Díaz-Arribas, Juan Antonio Valera-Calero, Marcos José Navarro-Santana, Juan Izquierdo-García, Rosa María Ortiz-Gutiérrez, Gustavo Plaza-Manzano

**Affiliations:** 1 Department of Radiology, Rehabilitation and Physiotherapy, Faculty of Nursery, Physiotherapy and Podiatry, Complutense University of Madrid, 28040 Madrid, Spain; josejalo@ucm.es (J.J.L.-M.); juavaler@ucm.es (J.A.V.-C.); marconav@ucm.es (M.J.N.-S.); juaniz02@ucm.es (J.I.-G.); rosaorti@ucm.es (R.M.O.-G.); gusplaza@ucm.es (G.P.-M.); 2 Faculty of Life and Natural Sciences, Nebrija University, 28015 Madrid, Spain; 3 Grupo InPhysio, Instituto de Investigación Sanitaria del Hospital Clínico San Carlos (IdISSC), 28040 Madrid, Spain; 4 Multidisciplinary Cardiac Rehabilitation Unit, University Hospital 12 de Octubre, 28041 Madrid, Spain

**Keywords:** chronic pain, low back pain, physiotherapy, therapeutic exercise, telerehabilitation

## Abstract

Low back pain (LBP) is a significant global health challenge due to its high prevalence, and chronicity and recurrence rates, with projections suggesting an increase in the next years due to population growth and aging. The chronic and recurrent nature of LBP, responsible for a significant percentage of years lived with disability, underscores the need for effective management strategies, including self-management strategies advocated by current guidelines, to empower patients and potentially improve healthcare efficiency and clinical outcomes. Therefore, the aim of this study was to analyze the added value of face-to-face visits in patients with chronic LBP undergoing a self-management program based on therapeutic exercises on pain intensity, disability, quality of life and treatment adherence and satisfaction. A randomized clinical trial was conducted, allocating 49 patients into a experimental group with a mobile health (mHealth) app usage and face-to-face sessions and 49 patients into an active control group without face-to-face sessions. Pain intensity, disability and quality of life were assessed at baseline, 4 weeks postintervention and 12 weeks postintervention. Patients’ satisfaction and adherence were assessed at the end of the study. The multivariate general model revealed no statistically significant time × group interaction for any outcome (*p* > 0.0068) but mental quality of life (*p* = 0.006). Within-group differences revealed significant improvements for all the clinical indicators (all, *p* < 0.001). Patients allocated to the experimental group reported greater satisfaction and adherence (both, *p* < 0.001) compared to the control group. The use of mHealth apps such as Healthy Back^®^ as part of digital health initiatives may serve as a beneficial approach to enhance the management of LBP.

## 1. Introduction

Low back pain (LBP), defined as “pain and discomfort, localized below the costal margin and above the inferior gluteal folds, with or without leg pain” [1], is currently one of the most important challenges faced by health care professionals. LBP prevalence and its resultant disability have been meticulously documented in the Global Burden of Disease Study 2021, which provides a granular view of LBP’s impact over a 30-year period. In 2020, LBP affected an estimated 619 million people globally. Despite a slight decrease in age-standardized rates over the past three decades, the absolute number of individuals suffering from LBP has continued to rise, primarily due to population growth and aging, particularly in regions like Asia and Africa [2]. In addition, this study forecasted for 2050 a 36.74% increase in the total number of cases of LBP, expecting to affect around 843 million individuals worldwide [2].

In addition to this prevalence problem, its disabling nature is another important concern to be considered. Since LBP is commonly associated with persistent pain, psychological impairments and limited mobility, patients report a significant disability and a significant reduction in the ability to perform work-related tasks (which leads to absenteeism and decreased productivity) [3]. Consequently, LBP represents a worrying economic burden, not only due to direct healthcare costs but also because of indirect costs such as lost earnings and reduced productivity [4]. For instance, in the USA, chronic LBP accounts for an average of 10.75 lost workdays per year per person in the workforce, equating to approximately 264 million workdays lost annually [2]. This burden is not exclusive to high-income countries; in Brazil, an average of 100 days absent from work per person per year is due to LBP, with productivity losses equating to a significant portion of the cost attributed to the condition [2].

LBP chronicity and recurrence rates are also serious concerns as previous reports declared that up to 65% of patients reporting an acute episode of pain would suffer +1 episodes within the next 12 months [5], and up to 40% of the episodes can last for more than 6 weeks [6]. Since LBP is responsible for 7.7% of all years lived with disability (YLDs), clinical studies developing effective strategies for managing patients with LBP are needed.

These factors collectively underscore why LBP remains a leading cause of disability, necessitating integrated and early return-to-work interventions, and highlighting the urgent need for more high-quality, primary country-level data on both prevalence and severity to improve the accuracy of current estimates and to inform global strategies aimed at reducing the number of new episodes of LBP and its associated disability. In fact, current recommendations encouraged self-management strategies [7]. These approaches empower patients to take an active role in managing their pain, which is crucial given the chronic and recurrent nature of LBP [8]. Self-management techniques are aligned with current guidelines that advocate for nonpharmacological first-line treatments, such as exercise and physical therapy, which are essential components of a comprehensive LBP management plan [9]. The primary care setting is often the first point of contact for patients with LBP, where they can be educated about their condition and introduced to self-management techniques. This initial education is vital, as it sets the foundation for patients to understand that they can exert control over their pain and its impact on their lives [10]. In addition, self-management leads in multiple advantages such as increased healthcare system efficiency (as patients engaged in self-management programs may have less frequent need for clinical interventions and reduce the need of healthcare visits and associated costs [11]) or better prognosis in clinical outcomes (as patients adhered to self-management programs [12,13]).

Recent studies have highlighted the enhanced usage of mobile health apps (mHealth) in augmenting patient engagement and treatment efficacy. Investigations focusing on app-based engagement and health improvement have demonstrated encouraging outcomes among patients with diabetes [14,15,16], hypertension [17], cardiac pathologies [18] and in daily pain management without external assistance, as exemplified by the Keele Pain Recorder app [19].

Compelling evidence supports the efficacy of apps in prescribing exercise regimens for individuals suffering from lower back pain (LBP), as detailed in the studies by Chhabra et al. [20]. These studies advocate for daily aerobic programs combined with seven specific exercises targeted at LBP treatment, positioning health mobile apps as a vital instrument in managing chronic conditions.

Despite the positive trends towards improved engagement through app usage, there are notable limitations. These include the lack of reinforcement that face-to-face consultations provide and the invaluable insights that health professionals can contribute to the development of mobile apps, which could significantly enhance the outcomes [21].

Therefore, the main objectives of this study were to analyze the added value of face-to-face visits in patients with chronic LBP undergoing a self-management program based on therapeutic exercises tracked with a mobile phone app by comparing pain intensity, LBP-related disability, quality of life and treatment satisfaction differences between groups; within-group differences after 12 weeks and adherence rates differences between both groups. Based on the literature exposed previously, we hypothesize that patients with LBP participating in a self-management program that integrates both mobile health app-based therapeutic exercises and face-to-face consultations will experience greater improvements in pain intensity, LBP-related disability, quality of life and treatment satisfaction compared to those who solely rely on the app-based program. Furthermore, we anticipate that this integrated approach will yield higher adherence rates and more pronounced within-group improvements after 12 weeks, underscoring the value of combining digital health tools with traditional clinical support in managing chronic LBP.

## 2. Materials and Methods

### 2.1. Study Design

A multicenter single-blinded randomized controlled clinical trial, with two parallel groups (one experimental group and one active control group) was conducted to compare the effects of a therapeutic exercise program designed for patients with chronic LBP with and without regular face-to-face supervision on pain intensity, disability, quality of life and treatment satisfaction. The full protocol was prospectively registered and is open and accessible at ClinicalTrials.gov (registered 7 November 2022 and accessed on 10 November 2023, ID: NCT04975568).

In order to enhance the quality of the manuscript, this report followed the Consolidated Standards of Reporting Trials (CONSORT) [22] and the Enhancing the QUAlity and Transparency Of health Research (EQUATOR) [23] guidelines. The authors considered all the recommendations disclosed in the Declaration of Helsinki and the study protocol and design was carefully supervised and approved by two local Clinical Ethics Committees: Hospital Clínico San Carlos (ID: 19/514-E_Tesis), Hospital 12 de Octubre (ID23/317) and Health Canary Service (ID 2021-425-1). In addition to the signed a written informed consent describing the purpose of the study, the data required, their rights and all the details about the interventions prior to the data collection, all participants filled out a standardized document for collecting demographic data (i.e., age, gender, professional situation, professional activity), information about their routinary physical activity per week, and pharmacological treatments during the previous 12 months.

### 2.2. Participants

Between May and December 2021, a consecutive sample of patients reporting LBP were screened for potential eligibility criteria from two Hospitals located in Madrid and Las Palmas de Gran Canaria (Spain). General inclusion criteria were being aged between 18 to 65 years old and suffering chronic nonspecific low back pain for +12 weeks (this aligns with current classifications [24] that define the stages of a condition as acute during the initial 6 weeks, subacute if it persists for 6 to 12 weeks and chronic if it continues for 12 weeks or more), with a minimum pain intensity of 3 out of 10 points in the Numeric Pain Rating Scale and at least 20% of disability scored in the Oswestry Low Back Pain Disability Questionnaire. The rationale for including patients in chronic stages was based on the LBP chronicity and recurrence rates stated in the introduction. The exclusion criteria considered in this study were presence of red flags (symptoms, signs or findings that suggest the possibility of a serious or life-threatening condition that requires immediate attention), pregnancy and lactation, history of previous relevant trauma, back surgeries, balance impairments, visual dysfunctions, neurological conditions and inability to read, understand or complete questionnaires, verbal instructions or mobile phone apps. The age of the participants, the evolution time of their pathology, their weekly physical activity habits, medication intake and the physical characteristics demanded by their jobs were analyzed.

### 2.3. Sample Size Estimation

The G*Power software v.3.1.6. (Dusseldorf, Germany) for Mac OS was used to provide the minimum sample size estimation required to obtain acceptable statistical power. An a priori ANOVA test (repeated measures, within and between interaction) was run to compute the required sample size providing the desired level of significance, statistical power and effect size. Considering that Cohen determined an effect size of moderate magnitude (f = 0.25) to detect clinically relevant differences [25], the input parameters were set at α = 0.05, β = 0.05 (95% power), f = 0.25, 2 groups and 4 measurements. Considering that 36 participants (*n* = 18 per group) would be required for this statistical power and the longitudinal nature of the study, an additional 10% per measurement was added (*n* = 2 per group) for preventing potential losses. Therefore, at least 52 participants were required (*n* = 26 per group).

### 2.4. Interventions

#### 2.4.1. Self-Management Protocol: Therapeutic Exercise and Mobile Phone App Registry

**Figure 1:**
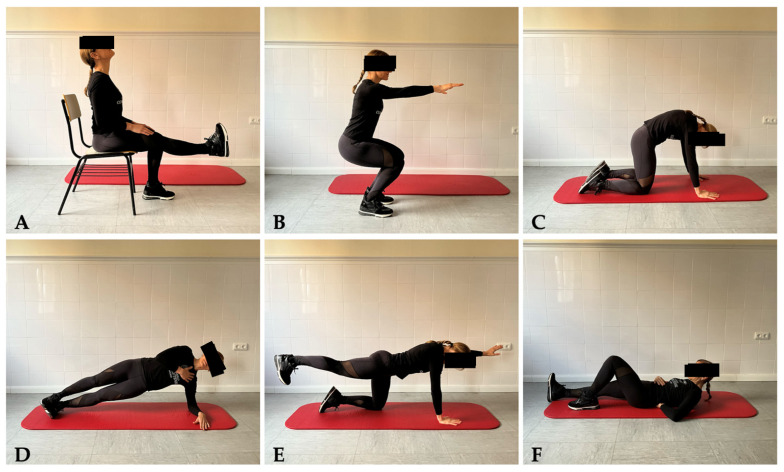
Illustrative photograms of instructive videos for each exercise.

The therapeutic exercise protocol was applied in this study for both groups and is based on the foundational “Big Three” exercises as delineated by McGill and Stuart [26]. This original program was adapted to include a suite of six exercises, comprising three for warm-up and three focusing on the strength and motor control. Four distinct programs (Basic, Intermediate, Advanced and Expert) were developed, each with progressively challenging exercises tailored to enhance the participants’ proficiency over time. Participants were instructed to select the most challenging level, but ensuring the correct performance of the exercises, allowing them using different levels for each exercise. All participants were instructed using visual aids collected in a PDF document as illustrated in Figure 1, and videos posted by the research team in a Youtube private link (only accessible through the app) for improving the execution fidelity and replication of the exercises.

A succinct description of the six exercises is as follows:

*Warm-up 1*: Seated Mobilization. Participants performed a smooth, combined movement of knee flexion while looking down and extension while looking up. The movement had to be performed slowly, dynamically and fluidly (Figure 1A).

*Warm-up 2*: Squat. Participants performed a partial squat using a chair as a reference point for the final position, synchronizing knee flexion with the elevation of the arms forwarded. The movement had to be executed in a dynamic, slow and fluid manner (Figure 1B).

*Warm-up 3*: Quadruped Spinal Mobilization. In a quadruped position, participants had to bring the gaze towards the navel, arching the back, and then reverse the curve dynamically, slowly and fluidly (Figure 1C).

*Exercise 1*: Side Bridge. Lying on one side with the legs straight and one foot in front of the other, participants were asked to place the elbow directly under the shoulder and the hand of the upper arm against the torso. Then, they had to elevate the pelvis to form a continuous line with the trunk and maintain this position for 7 to 10 s, ensuring the pelvis does not drop (Figure 1D).

*Exercise 2*: Bird Dog. Starting in a quadruped position with hands under shoulders and knees under hips, participants maintained a neutral spine while lifting one leg, attempting to extend it in line with the trunk, and simultaneously reaching the opposite arm forward (holding this position for 7 to 10 s as shown in Figure 1E).

*Exercise 3*: Curl Up. Lying on the back with one leg supported and bent, and the other leg bent at 90 degrees in the air “pulling” the toes towards oneself, participants had to maintain a small gap at the lumbar spine. Then, they had to perform abdominal contractions lasting 7 to 10 s, attempting to draw the ribs and pubis closer together, and lifting the head off the ground with the intention of elongating the spine (Figure 1F).

**Figure 2:**
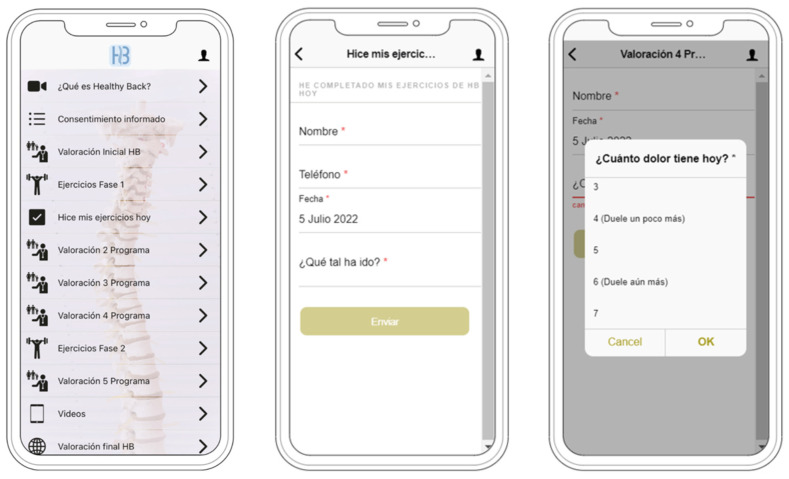
Healthy Back ^®^ app interface.

Participants were instructed to adhere to the exercise sequence three times weekly over a period of twelve weeks (50 min each session). The Healthy Back App^®^ (v.1.0.) was meticulously crafted to furnish users with access to all previously outlined materials, to document self-reported patient outcomes and their exercise adherence. Furthermore, the app incorporated a sophisticated chat interface, enabling patients to conduct remote consultations with their physiotherapist for the resolution of queries related to the exercise program, individual health concerns or to request additional support through video calls or teleconsultations, ensuring a more nuanced and interactive explanation when necessary. Sample images of the app can be visualized in Figure 2.

#### 2.4.2. Face-to-Face Sessions

Participants allocated into the experimental group attended an in-person health education and therapeutic exercise session biweekly, completing a total of six face-to-face sessions throughout the intervention process. Every session have a duration of 45 min and was a session group-based with 6 participants. As part of the program’s follow-up protocol, a reminder of the upcoming session (date and time) and the necessary assessment questionnaires to be filled out for ongoing treatment were sent to participants 48 h before each in-person session.

*Session 1* involved learning the exercises and their adaptations based on the individual characteristics of each participant. There was also an introductory exploration of the anatomy and biomechanics of the spine directly related to the program’s exercises.

In *Session 2*, a review of the exercise techniques was conducted, teaching adaptations if participants had experienced difficulties, and progressions were introduced based on the feedback provided through the app. A brief health education class was also held to emphasize the importance of breathing as a complement to the exercises.

*Session 3* repeated the review process from Session 2 to continue the personalization of the exercises. The theoretical content covered the significance of pelvic floor physiotherapy and its direct connection to chronic lower back pain. The session not only offered specific exercise recommendations but also reinforced the knowledge from the previous session about the importance of breathing in direct relation to the pelvic floor.

*Session 4* focused on the review and personalization of the exercises. A discussion about pain perception was shared among all group participants to facilitate learning through a “patient school” approach, which helps individuals normalize their condition based on the experiences of others with similar clinical profiles.

In *Session 5*, the usual review was conducted to maintain the individualization of the program. The “patient school” concept introduced in Session 4 continued, with this session aiming to educate on the impact of the condition on the quality of life. It included brainstorming on how to manage the pathology in daily life activities with the goal of normalizing the clinical presentation of the pathology.

*Session 6* involved a review of the exercises as in all previous sessions. This final session added a theoretical component, offering recommendations for continuing the practices undertaken during the 12-week Healthy Back^®^ program.

### 2.5. Randomization and Masking

Subjects from both hospitals were allocated to either the experimental group (receiving face-to-face sessions and a self-management exercise-based protocol) or to the active control group (not receiving these sessions, but undergoing the same exercise protocol), through a process of random selection using a random number generating software (Research Randomizer, version 4.0). Cards indicating the group assignment were individually numbered, folded and placed in sealed envelopes that were not see-through to ensure the assignments remained undisclosed. An independent researcher chose an envelope and carried out the assignment process. The details of the group assignments were disclosed to the lead researcher only after the initial data had been gathered. The evaluator was not aware about the group assignments during the outcomes’ measurement and analysis.

### 2.6. Primary Outcome

#### Pain Intensity

Pain intensity was considered the primary outcome for this study. The instrument used for assessing LBP intensity was the Numeric Pain Rating Scale (NPRS) as previous reports reported excellent test–retest reliability (Intraclass correlation coefficient > 0.9) and acceptable accuracy (minimum detectable changes of 0.10 points and standard error of measurement of 0.04 points) and responsiveness (minimal clinically important difference of 1.71 points) [27].

Patients were asked to select a number between 0 (complete absence of pain) and 10 (worst pain imaginable) to represent their pain severity. In order to improve the accuracy of the measurements, patients were asked to rate (1) their current pain intensity; (2) the worst pain intensity they suffered during the previous 7 days and (3) the lowest pain intensity suffered during the previous 7 days. The mean average of these 3 scores was calculated and used for the analyses [28].

### 2.7. Secondary Outcomes

#### 2.7.1. Pain-Related Disability

The evaluation of LBP-related disability was conducted using the Oswestry Low Back Pain Disability Questionnaire (ODI), which has been shown to have satisfactory reproducibility [29]. This self-administered questionnaire is composed of ten items, with responses measured on a 6-point Likert scale. On this scale, a score of 0 indicates “no disability,” while a score of 5 signifies “extreme disability.” The final score is calculated as a percentage, derived by doubling the sum of the scores from the questionnaire. The level of a participant’s disability is categorized as minimal, moderate, severe, crippled or complete, corresponding to final scores of 0–20%, 21–40%, 41–60%, 61–80% and 81–100%, respectively [30].

#### 2.7.2. Quality of Life

Patients’ quality of life was assessed using the Short Form Health Survey-12 (SF-12). This instrument was used instead of the SF-36 since respondents requires less than a third of the usual time needed to complete this short version and both instruments are almost perfectly correlated [31]. Both the physical and mental component summaries were calculated, each one with scores ranging between 0 and 100 (with higher scores denoting an improved quality of life [31].

#### 2.7.3. Treatment Adherence and Satisfaction

Treatment adherence was assessed using the Healthy Back^®^ app as it tracked the number of sessions completed by each participant.

Patient’s satisfaction treatment was assessed using the validated Spanish version of the Patient Satisfaction Questionnaire (PSQ-E) [32]. It is also a self-reported questionnaire which consists of a 14-item battery and responses in a 6-points scale where 1 is considered “bad satisfaction” and 5 is consider “excellent satisfaction” with a neutral value according to “I don’t Know”. Final scores ranges between 0 and 70, and scores over 20 points are defined as a good global satisfaction. It is possible also analyze every item alone and when the evaluation is under 75% in the values “excellent”, “Very good” or “good” the questionnaire shows an improvement area, when it is between 75% and 90% the questionnaire shows a neutral area and when it is over 90% it is an excellence area.

### 2.8. Treatment Side Effects

All participants were instructed to record any negative incidents or aftereffects, defined as symptoms deemed intolerable by the patient or those necessitating additional medical intervention, occurring during or subsequent to the treatment period (spanning the one-month timeframe of the study) [33].

### 2.9. Statistical Analysis

The Statistical Package for the Social Sciences (SPSS v.29.1.1, Armonk, NY, USA) was employed on the Sonoma Operating System (Mac OS v.14.0) to run all statistical computations, adopting a significance demarcation of *p* < 0.05. Data distribution was evaluated via histogram inspection and application of the Shapiro–Wilk test to continuous variables. A *p*-value inferior to 0.05 was indicative of a non-Gaussian distribution, whereas a *p*-value superior to 0.05 suggested normal distribution. Descriptive statistical methods delineated the characteristics of the sample. In the context of categorical variables, frequency and relative frequency for each category were reported (e.g., the number and percentage of female and male subjects and participants allocated to each group). Pertaining to continuous variables, indices of central tendency (the mean for Gaussian variables and the median for non-Gaussian variables) were employed alongside dispersion metrics (standard deviation for Gaussian-distributed variables and interquartile range for variables divergent from Gaussian distribution).

Differential analysis between groups was conducted utilizing multivariate linear general models, which incorporated pain intensity, disability and quality of life as dependent variables. The models accounted for group classification (experimental and control groups) and time (baseline, 4 weeks postintervention and 12 weeks postintervention) as primary factors, considering baseline scores as adjusting covariates. To address the issue of multiple comparisons, post hoc analyses incorporating the Bonferroni correction were executed (group × time). The magnitude of the observed effects was quantified using the partial eta squared ($\eta_{p}^{2}$), with values of 0.01, 0.06 and 0.14 representing small, medium and large effect sizes, respectively. A revised significance level of *p* < 0.00625 (0.05/8) was established to account for the multiple testing scenario [34]. Finally, Student t-tests were used to assess between-group differences regarding the treatment adherence and satisfaction at the end of the treatment.

## 3. Results

**Figure 3:**
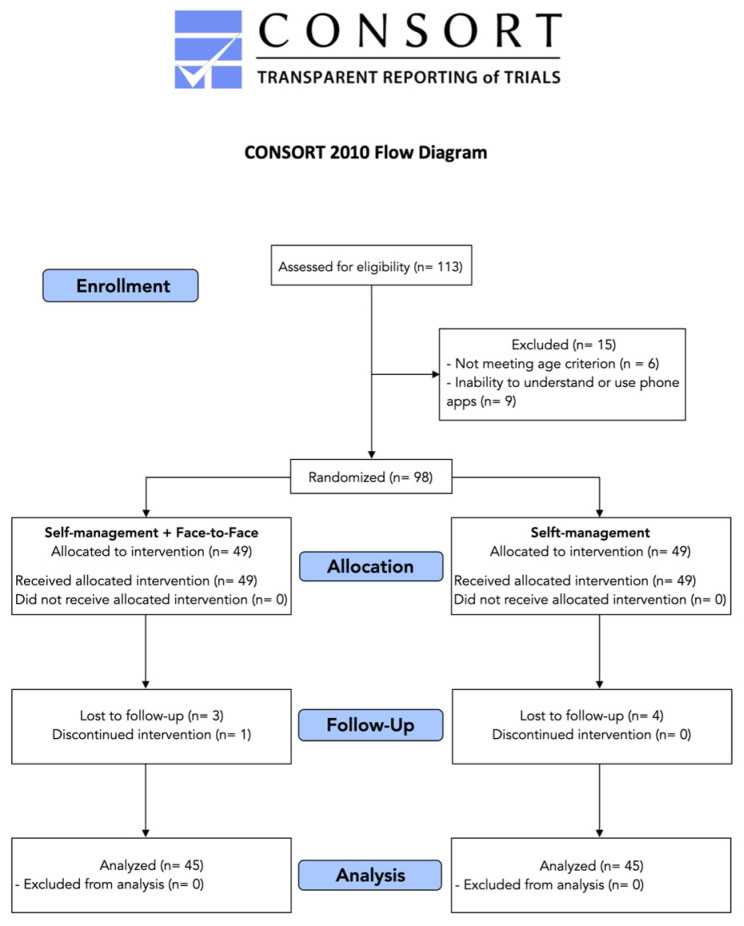
CONSORT diagram describing the participants’ flowchart.

From 113 subjects initially screened for eligibility responding to the announcement, fifteen participants (*n* = 15) were excluded from the study because they did not meet the age range stablished (*n* = 6) and report inability to understand and use mobile phones apps (*n* = 9). Therefore, ninety-eight participants (*n* = 98) were finally included and randomized to both groups (each group, *n* = 49). The participants’ flow chart diagram is illustrated in Figure 3.

Sociodemographic data and clinical characteristics by intervention group are described in Table 1. At baseline, both groups showed comparable baseline demographic characteristics as *p* values (all, *p* > 0.05) indicate that both groups were balanced in terms of gender, weekly physical activity, professional situation and LBP chronicity. In addition, the medication intake analyses revealed no significant differences at baseline (*p* = 0.821) between the experimental group (EG) and the control group (CG): analgesics (EG 20.6%; CG 14.3%), muscle relaxants (EG 5.9%; CG 10.7%), other drugs (EG 2.9%; CG 3.6%), a combination of analgesics and other drugs (EG 14.7%; CG 28.6%), a combination of muscle relaxants and analgesics (EG 35.3%; CG 28.6%), a combination of anti-inflammatories and relaxants (EG 5.9%; CG 7.1%), a combination of analgesics, muscle relaxants and other drugs (EG 11.8%; CG 7.1%) and anti-inflammatories, analgesics, relaxants and other drugs (EG 2.9%; CG 0.0%). Regarding the dosage, no significant differences were found between groups (*p* = 0.359): medication intake on an occasional basis (EG 38.2%; CG 50.0%), prescribed dosage (EG 50.0%; CG 32.1%) and exceeding the prescribed dosage (EG 11.8%; CG 17.9%).

Time (baseline, after 4 weeks postintervention and after 12 weeks postintervention) and group (experimental and control) comparisons data regarding pain intensity, quality of life and pain-related disability are accessible in Table 2. At baseline, both groups demonstrated comparable pain intensity (mean difference 0.74; 95% CI −0.11 to 1.60; *p* = 0.059), physical (mean difference 0.77; 95% CI −3.8 to 4.8; *p* = 0.705) and mental (mean difference 1.77; 95% CI −2.8 to 6.3; *p* = 0.436) quality of life and pain-related disability (mean difference 0.27; 95% CI −6.7 to 7.2; *p* = 0.934) as expected after patients’ randomization. Group * Time interaction analyses revealed no significant differences for pain intensity, physical quality of life or pain-related disability (all, *p* > 0.0068). However, mental quality of life showed statistically significant differences (*p* = 0.006).

Results also showed no significant differences analyzing group differences (all, *p* > 0.006). However, time interactions revealed significant improvements for all the outcomes analyzed (all, *p* < 0.01). Effect sizes for each analysis are also reported in Table 2. None of the participants reported adverse effects during the study.

Table 3 reports data about the treatment satisfaction and patients’ adherence. The results obtained revealed statistically significant differences between both groups for the number of self-management sessions completed (*p* < 0.001) and their satisfaction at the end of the study (*p* < 0.001), indicating a greater number of sessions completed and treatment satisfaction in the experimental group compared to the control group.

Finally, the intervention effects on the medication consumption revealed no significant differences between both groups (*p* = 0.371): Although in EG 20.6% and CG 46.4% no medication intake reduction was declared, EG 17.6% and 21.4% declared analgesics intake reduction, EG 5.9% and CG 10.7% declared muscle relaxants intake reduction, EG 20.6% and 7.1% reduced the consumption of anti-inflammatories, EG 8.8% and CG 7.1% reduced the consumption of anti-inflammatories and relaxants, EG 5.9% and CG reduced the consumption of analgesics and anti-inflammatories, EG 2.9% reduced the consumption of analgesics, relaxants and other drugs, EG 2.9% and CG 3.6% reduced the consumption of analgesics, anti-inflammatories and other drugs, EG 5.9% and CG 3.6% reduced the consumption of analgesics, anti-inflammatories and relaxants and EG 2.9% reduced the consumption of analgesics, anti-inflammatories, relaxants and other drugs.

## 4. Discussion

This study aimed to analyze the impact of face-to-face supervision sessions on the effectiveness of self-management therapeutic exercise programs in patients with chronic LBP. Although there are several studies investigating the utility of mHealth in chronic conditions for improving the communication between patients and health professionals, collecting and monitoring changes over the time or as a treatment option [35,36,37,38], limited evidence is available for this specific population and exercise-based interventions.

This study corroborates that regular exercise in a home-based program significantly reduces pain, with further reductions observed when face-to-face supervision sessions are added. These findings align with the existing literature [39,40,41]. However, it is noteworthy that some studies reported less pronounced improvements [40,41], possibly due to participants starting with lower pain levels [39,41,42] or the postintervention follow-up duration being shorter [39]. Albadalejo et al. [43] reached similar conclusions, noting that incorporating health education classes altered subjective pain perception.

Contrastingly, certain studies found no significant difference between remote face-to-face treatment and app-based remote treatments [20,44], or even reported superior outcomes with remote exercise programs specifically designed for LBP patients [45]. The most significant contribution of this study is the incorporation of biweekly sessions in the remote assistance framework, leading to more substantial changes in pain levels.

Regarding differences in disability between groups, this study found no significant variations. The results in this aspect remain a subject of debate in the literature, largely influenced by the baseline characteristics of the subjects. Studies where baseline scores on the ODI were below 40 points did not demonstrate statistically significant results [44,46,47]. Conversely, in cases where patients had baseline scores above 40 points, the effectiveness of the program was statistically significant [42,48].

On the other hand, a recent review [49] examined the effects of using 5 mHealth apps for LBP self-management reported in seven studies. Most studies reported promising results, with 86% showing a reduction in pain and 75% showing improved disability levels. However, there was heterogeneity in app types, pain duration and comparison groups. The content of the apps mainly included therapeutic exercises for strength, mobility and mindfulness. The use of apps may be a valuable addition to LBP self-management, as some studies reported clinically meaningful pain reduction within 6 to 16 weeks and significant improvements in functional ability. However, the authors recognized important limitation that this study tried to address.

For example, most of mHealth studies included in the revision had important flaws regarding the methodological quality and risk of bias. In this study, the PEDro scale was checked during the study design stage in order to address these methodological issues [50]. However, patients and therapists’ blinding are still limited in these designs. In addition, this revision reported important concerns regarding the generalizability of findings, as some high-sample studies failed to report pain duration or type, patients’ satisfaction or app use. For addressing these flaws, this study included this information. Even if both groups passed the threshold (20 points out of 70 [32]) to consider the intervention as “satisfactory”, the general satisfaction was significantly better in the group who assisted to the face-to-face sessions.

Treatment adherence is considered an essential factor for the medium and long-term results in patients with chronic pathologies [51]. In fact, Mannion et al., [52] conducted a prospective study evaluating the association between patients’ adherence and outcome results during a program of spinal motor control exercises, finding a moderate correlation with pain reduction and disability. In line with our results, they found a very good adherence (85% of completed sessions) and clinical improvement associated.

Factors influencing adherence are multifaceted, involving patients (i.e., health insurance coverage, financial status, time constraints, high pain intensity and willingness to complete questionnaires), therapists (i.e., experience with successful treatments not recommended in the guideline, knowledge from specific courses or training, time constraints and physiotherapist’s own satisfaction with treatment outcomes), guideline characteristics (lack of information about psychosocial prognostic factors and psychosocial treatment options, and the conflict between patient expectations and guideline recommendations), institutional (i.e., financial status of the practice, agreements with healthcare insurers and the average number of treatment sessions offered) and implementation factors (i.e., insufficient dissemination and training, leading to a lack of familiarity with the guidelines among physiotherapists) [53]. Therefore, for improving patients’ adherence, guidelines should provide more detailed information on psychosocial aspects and implementation should include effective training and communication strategies to manage patient expectations [54].

Finally, the review [51] declared small sample sizes and inadequate reporting of outcome results. This study provides a proper sample size calculation following the recommendations of the literature in order to obtain acceptable statistical power, and reported the statistical estimates of the tests used, confidence intervals, *p* values and effect size estimates. A similar designed study was conducted by Mbada et al., [55], comparing the effects of a telerehabilitation strategy based on McKenzie Therapy with face-to-face interventions. These authors set an alpha level at *p* < 0.05 for analyzing mean differences between two groups at three moments (baseline, 4 weeks postintervention and 8 weeks postintervention). Even if significance levels can be discussed and in line with our results, the authors reported no differences between groups for most of the outcomes assessed. Although in contrast with Mbada et al. [55], the self-management strategy in this study was based on McGill’s therapeutic exercises as this selection has been widely supported in the literature for managing patients with chronic LBP [56], multimodal approaches may enhance the outcomes results in the long term and improve the patients’ adherence and satisfaction.

### Limitations

Although the authors of this research tried to address methodological quality flaws reported in other studies, this investigation is not free of limitations and should be recognized for guiding future studies on this topic.

First, we did not include any cost evaluation effect in this study. Future research could include this information to estimate the economic impact of mixed care programs and provide additional support for these strategies. Secondly, psychosocial factors are closely related to the treatment success, patients’ adherence and satisfaction [39,45,57]. Future studies should consider the inclusion of these variables to analyze the impact of these strategies on catastrophism, kinesiophobia, depression and anxiety. Finally, this study was limited to a 12-week follow-up period. Further research is needed for observing long-term effects.

## 5. Conclusions

The implementation of digital health programs through mHealth apps could be an effective strategy for improving pain intensity, quality of life and pain-related disability of patients with chronic LBP as the Healthy Back ^®^ app, designed based on McGill’s exercises, demonstrated significant clinical severity improvements. Although face-to-face sessions did not show additional benefit for most of the outcomes assessed (only mental quality of life showed better results in this group compared to only app usage), periodic presential sessions may enhance the patients’ satisfaction and adherence.

## Figures and Tables

**Table 1:** Baseline demographic data, routinary physical activity, professional situation and LBP duration comparison between groups.

	Experimental Group (*n* = 45)	Control Group (*n* = 45)	*p* Value
Demographic Information			
Females (%)	76.5	89.3	0.189
Age (years)	52.2 ± 9.8	49.9 ± 10.0	0.338
Weekly Physical Activity			
1 day a week (%)	29.4	25.0	0.923
2 days a week (%)	26.5	32.1	
3 days a week (%)	14.7	17.9	
+4 days a week (%)	29.4	25.0	
Professional Situation			
Working (%)	55.9	71.4	0.397
Sick Leave (%)	32.4	14.3	
Laboral inhability (%)	2.9	0.0	
Not applicable (%)	8.8	14.3	
Clinical Information			
Symptoms Duration (years)	3.2 ± 1.1	3.3 ± 1.3	0.646

**Table 2:** Differential analyses between groups for pain intensity, quality of life and pain-related disability.

	Experimental Group (*n* = 45)			Control Group (*n* = 45)			Between-Group Differences		
	Baseline	4 Weeks	12 Weeks	Baseline	4 Weeks	12 Weeks	Group × Time	Group	Time
Pain Intensity									
NPRS (0–10)	7.0 ± 1.8	4.1 ± 2.0	4.3 ± 1.6	6.3 ± 1.5	4.7±1.6	4.6 ± 1.4	F = 2.818 $\eta_{p}^{2}$ = 0.030 *p* = 0.062	F = 0.073 $\eta_{p}^{2}$ = 0.000 *p* = 0.788	F = 35.084 $\eta_{p}^{2}$ = 0.280 *p* < 0.001
Quality of Life									
SF-12 Physical (0–100)	35.1 ± 8.4	41.0 ± 6.9	41.7 ± 6.4	35.8 ± 7.3	37.1 ± 5.9	38.2 ± 4.6	F = 2.124 $\eta_{p}^{2}$ = 0.023 *p* = 0.123	F = 4.555 $\eta_{p}^{2}$ = 0.025 *p* = 0.034	F = 7.912 $\eta_{p}^{2}$ = 0.081 *p* < 0.001
SF-12 Mental (0–100)	40.6 ± 10.4	41.4 ± 7.7	52.9 ± 5.9	40.1 ± 9.5	49.3 ± 6.5	53.2 ± 6.1	F = 5.301 $\eta_{p}^{2}$ = 0.056 *p* = 0.006	F = 4.775 $\eta_{p}^{2}$ = 0.026 *p* = 0.030	F = 40.068 $\eta_{p}^{2}$ = 0.308 *p* < 0.001
Pain-Related Disability									
ODI (0–100)	30.6 ± 16.3	20.8 ± 12.9	16.5 ± 13.7	29.8 ± 9.3	18.9 ± 13.3	18.1 ± 15.0	F = 0.254 $\eta_{p}^{2}$ = 0.776 *p* = 0.003	F = 0.005 $\eta_{p}^{2}$ = 0.942 *p* = 0.000	F = 14.637 $\eta_{p}^{2}$ = 0.140 *p* < 0.001

NPRS: Numeric Pain Rating Scale; ODI: Oswestry Disability Index.

**Table 3:** Patients’ treatment adherence and satisfaction evaluated at the end of the study.

	Experimental Group (*n* = 45)	Control Group (*n* = 45)	Difference
% Sessions Completed			
Face-to-Face (%)	100.0 ± 0.0	-	
Self-Management (%)	78.1 ± 13.1	64.4 ± 9.2	13.6 (7.8; 19.7) *p* < 0.001
PSQ (0–70)	47.7 ± 4.2	35.7 ± 2.9	12.0 (10.1;13.9) *p* < 0.001

Baseline scores are mean and standard deviation. Between-group differences are reported as mean difference (95% Confidence Interval) and *p* Values.

## Data Availability

Data are contained within the article.
